# Three Dimensional Finite Element Analysis to Detect Stress Distribution in Spiral Implants and Surrounding Bone

**Published:** 2009

**Authors:** Matteo Danza, Annalisa Palmieri, Francesca Farinella, Giorgio Brunelli, Francesco Carinci, Ambra Girardi, Giuseppe Spinelli

**Affiliations:** * Senior Lecturer, School of Dentistry, University of Chieti, Chieti, Italy; ** Post Doctoral Fellow, Department of Maxillofacial Surgery, University of Ferrara, Ferrara, Italy; *** Doctoral Fellow, Department of Maxillofacial Surgery, University of Ferrara, Ferrara, Italy; **** Senior Lecturer, Department of Maxillofacial Surgery, University of Ferrara, Ferrara, Italy; ***** Associate Professor, Department of Maxillofacial Surgery, University of Ferrara, Ferrara, Italy; ****** Doctoral Fellow, Department of Histology, Embryology and Applied Biology, University of Bologna, Bologna, Italy; ******* Consultant, Department of Maxillofacial Surgery, Careggi Hospital, Firenze, Italy

**Keywords:** Biomechanics, Dental stress analysis, Finite element analysis, Spiral implant

## Abstract

**Background:**

The aim of research was to study spiral family implant by finite element analysis (FEA) inserted in different bone qualities connected with abutments of different angulations.

**Methods:**

The biomechanical behaviour of 4.2 × 13 mm dental implants, connecting screw, straight and 15° and 25° angulated abutments subjected to static loads, in contact with high and poor bone qualities was evaluated by FEA.

**Results:**

The lowest stress value was found in the system composed by implants and straight abut-ments loaded with a vertical force, while the highest stress value was found in implants with 15° angulated abutment loaded with an angulated force. In addition, we found the lower the bone quality, the higher the distribution of the stress within the bone.

**Conclusion:**

Spiral family implants can be used successfully in low bone quality but applying a straight force is recommended.

## Introduction

**Figure 1:**
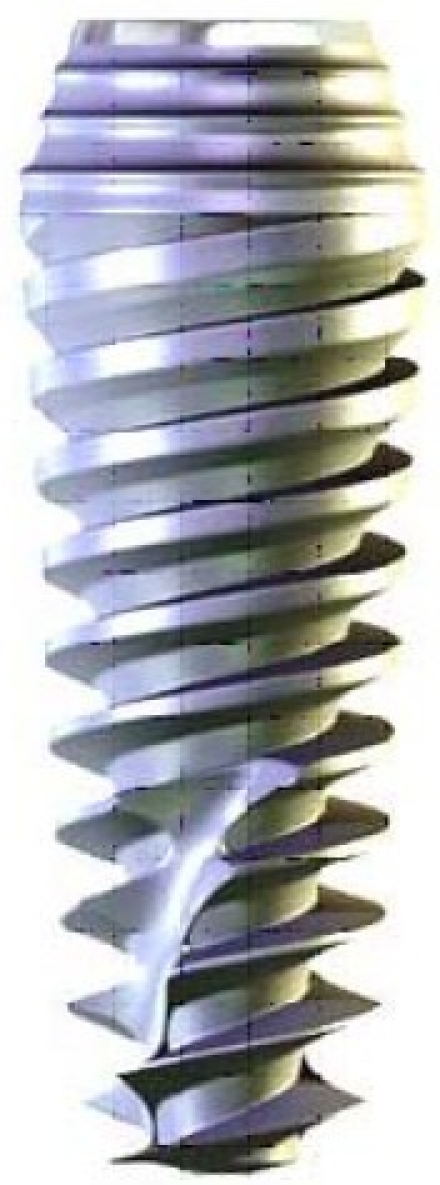
A spiral family implant.

The biomechanical behavior of an osseointegrated dental implant plays an important role in its functional longevity inside the bone. Finite element analysis (FEA) has been used extensively to predict the biomechanical performance of various dental implant designs as well as the effect of clinical factors on implant success. By understanding the basic theory, method, application, and limitations of FEA in implant dentistry, the clinician will be better equipped to interpret the results of FEA studies and extrapolate these results to clinical situations.^1^ FEAs have been used to study the effects of various shapes of dental implants on distribution of stresses generated in the surrounding jaw bone and to determine an optimal thread shape for better stress distribution.^2^ It has been hypothesized that marginal bone resorption may be resulted from accumulation of microdamages in the bone. In light of this, a dental implant should be designed in such a shape that the peak of stresses arising in the bone are minimized. The load on an implant can be divided into vertical and horizontal components. In earlier studies, it was found that the peak bone stresses resulting from vertical load components and those resulting from horizontal load components arise at the top of the marginal bone, and that they coincide with spatially. These peak stresses are added together and produce a risk of stress-induced bone resorption.^3^ In addition, creation of an appropriate alignment between forces and implant long axis are vital for its long-term success. Excessive load generated around an inclined implant causes micro-cracks in bone, which in turn leads to implant loosening and eventual failure.^4^ Using FEA, it was shown that, with a conical implant-abutment interface at the level of marginal bone, in combination with retention elements at the implant neck, and with suitable values of implant wall thickness and modulus of elasticity, the peak bone stresses resulting from an axial load arose further down in the bone. This means that they were spatially separated from the peak stresses resulting from horizontal loads. When the same implant-abutment interface was located 2 mm more coronally, these benefits disappeared. This also resulted in substantially increased peak bone stresses.^5^ Recently, a new type of implant with a spiral form has been produced (Officine Meccaniche di Precisione srl, Ferrara, Italy) (Figure 1).

We performed a three dimensional FEA study to analyze first, the stress distribution within two different bone types (i.e., high and poor qualities) due to forces applied to three implant systems (i.e., one spiral implant plus one straight abut-ment, or one 15° angulated abutment, or one 25° angulated abutment), and second, the stress distribution within the implant system (i.e., implant and abutment).

## Materials and Methods

**Figure 2:**
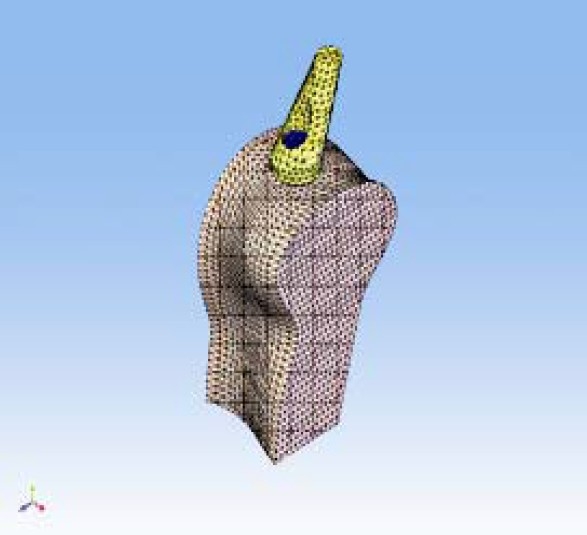
Mesh formation where the single finite elements were connected among them by nodes.

The biomechanical behaviour of an implant system (Officine Meccaniche di Precisione srl, Ferrara, Italy) subjected to static loading in contact with high (D1) and low (D4) density bone tissue was evaluated in the present study. The implants were 4.2 mm in diameter and 13 mm in length and abutments were straight and 15° and 25° angulated. FEA was used in order to determine strain distribution in the tissues around the implant related to different bone structure, abutment angulations and loading. It was important to specify the implant system (i.e., implant plus abutment), the kind of bone, the entity of axial and transversal loads applied to the different configurations in order to evaluate the biomechanical behavior. The directions of axial and transversal loads that stress implant and bone tissue when applied to the implant top were evaluated. A double system was analyzed: a) F_Y_ strength acting along Y axis and having 200 N intensity; b) F_Y_ and F_Z_ couple of strengths applied along the Y and Z directions and having respectively 200 N and 140N intensity. In order to plan the FEA and to reach the relative results, it was necessary to create mathematical models that are curves, surfaces and solids. Once drawn the systems that were object of the study by Computer Aided Design (CAD), the FEA discretized solids composing the system in many infinitesimal little elementary solids defined finite elements. This leads to a mesh formation where the single finite elements were connected among them by nodes. For the 3 unit bone-implant, about 19,000 nodes and about 105,000 tetrahedral elements having 10 parabolic nodes were employed. Once the solids, the mesh and the planned loads (direction and intensity) were defined, a definition of the chemico-physical properties of materials was needed. For biomechanical analyses of materials subjected to low intensity strengths, the materials can be considered homogeneous, linear and isotropic. Then, the FEA simulation was performed hypothesizing linearity between loads and deformations. The portion of bone containing the implant was bound around two sides by joints removing all degrees of freedom to the system (Figure 2).

**Figure 3:**
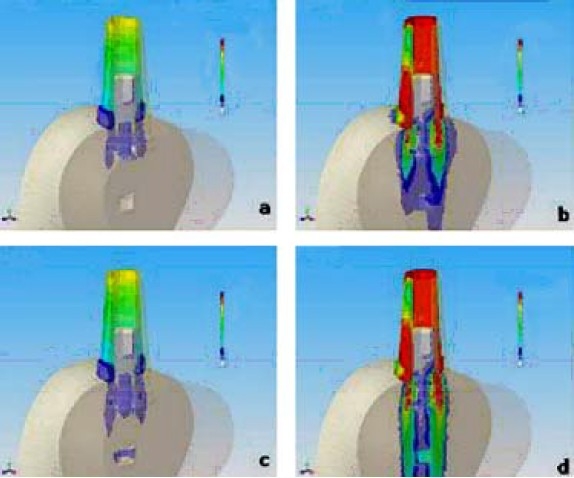
Stress distribution (Von Mises) of SFB implant connected with straight abutment. a) bone quality D1 and force Fy = 200 N b) bone quality D1 and force Fy = 200 N and Fz = 140 N c) bone quality D4 and force Fy = 200 N d) bone quality D4 and force Fy = 200 N and Fz = 140 N FNx01Colours from yellow to red indicate stress. The beige colour corresponds to zero or toward zero stress while the red colour indicates maximum stress.

**Figure 4:**
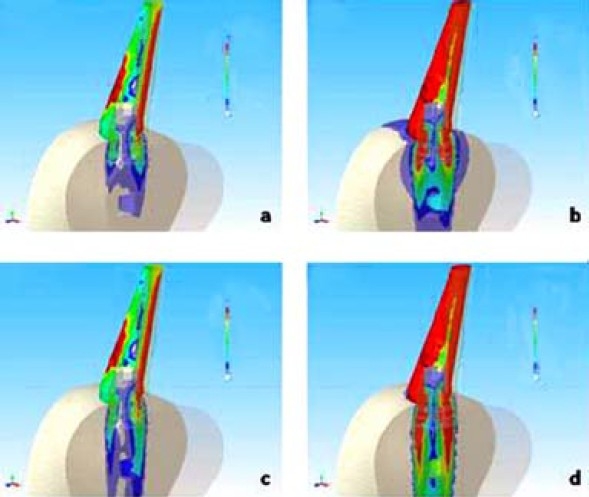
Stress distribution (Von Mises) of SFB implant connected with 15 degree abutment. a) bone quality D1 and force Fy = 200 N b) bone quality D1 and force Fy = 200 N and Fz = 140 N c) bone quality D4 and force Fy = 200 N d) bone quality D4 and force Fy = 200 N and Fz = 140 N FNx01Colours from yellow to red indicate stress. The beige colour corresponds to zero or toward zero stress while the red colour indicates maximum stress.

The pivot inside the bone tissue was bound by contact elements so as the connecting screw and the abutment. The CAD 3-D mathematical models used for FEA were realized using a surface modeller (RHINOCEROS 4.0 - McNeel Europe, Barcelona, Spain) and a solid modeller (SOLID WORKS 2007 SP2.2 – Solid Works Corporation Headquarters, Concord, MA), both belonging to WINDOWS XP Professional Edition-SP1-Microsoft Corporation, Milano, Italy). The discretization in finite elements and the FEA were realized by NEiFUSION 1.12 (Noran Engineering, Inc., Westminster, CA). The systems analyzed were as the following (Figures 3 and 4):

1. Implant having 4.2 mm diameter with 0° abutment: D1 bone and vertical strength.
2. Implant having 4.2 mm diameter with 0° abutment: D1 bone and tilted strength.
3. Implant having 4.2 mm diameter with 0° abutment: D4 bone and vertical strength.
4. Implant having 4.2 mm diameter with 0° abutment: D4 bone and tilted strength.
5. Implant having 4.2 mm diameter with 15° abutment: D1 bone and vertical strength.
6. Implant having 4.2 mm diameter with 15° abutment: D1 bone and tilted strength.
7. Implant having 4.2 mm diameter with 15° abutment: D4 bone and vertical strength.
8. Implant having 4.2 mm diameter with 15° abutment: D4 bone and tilted strength.
9. Implant having 4.2 mm diameter with 25° abutment: D1 bone and vertical strength.
10. Implant having 4.2 mm diameter with 25° abutment: D1 bone and tilted strength.
11. Implant having 4.2 mm diameter with 25° abutment: D4 bone and vertical strength.
12. Implant having 4.2 mm diameter with 25° abutment: D4 bone and tilted strength.

In Table 1, all the characteristic values of “E” Young’s Modulus and “v” Poisson Ratio have been reported.^6^–^14^

**Table 1:** The characteristic values of “E” Young’s Modulus and “v” Poisson Ratio.

Particular	Material	“E” Young’s Modulus (Pa)	“v” Poisson Ratio (dimensional)
Mandible section	D1 Cortical Bone	1.47E10 Pa	0.3
Mandible section	D4 Cortical Bone	0.14E10 Pa	0.3
Fixture	Titanium	1.05E11 Pa	0.35
Connecting screw	Titanium	1.05E11 Pa	0.35
Abutment	Titanium	1.05E11 Pa	0.35

## Results

The results obtained from the FEA simulation showed the relationship between loads applied on the system, geometrical characteristics of materials, joints and strain. One of the most frequent used theories for determining stress in bone matrix is “Von_Mises” theory. This theory was applied to this experiment in order to determine stress distribution at the bone-implant interface.

**Figure 5:**
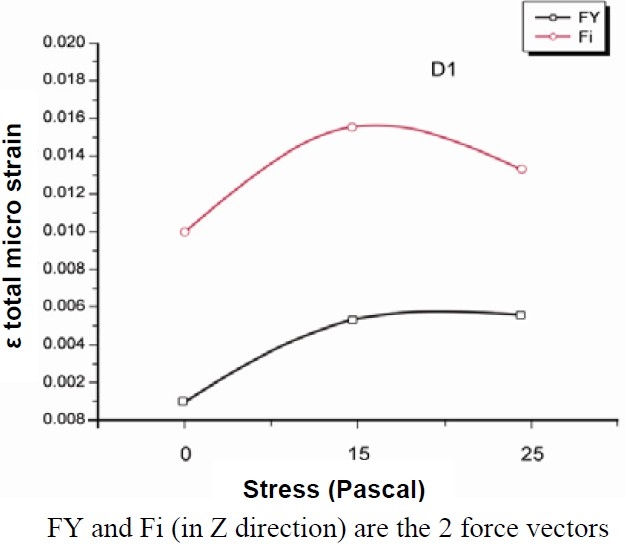
Values of stress distribution in bone of high quality.

**Figure 6:**
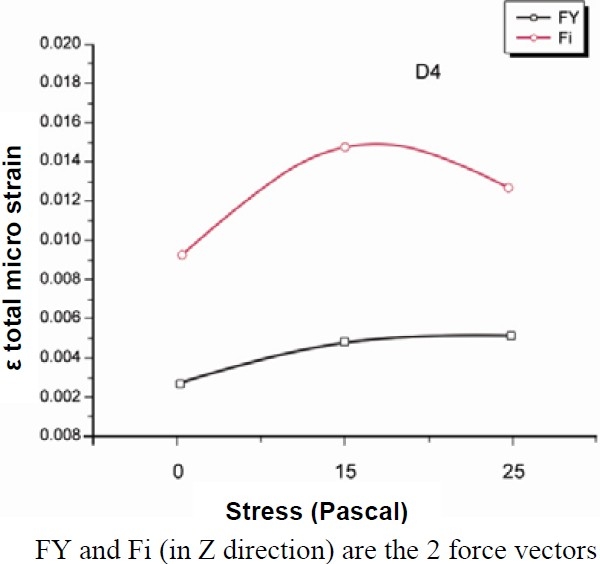
Values of stress distribution in bone of low quality.

The figures of the single systems (Figures 3 and 4) following the same nomenclature used in the list of materials and methods (section passing for X = 0 through YZ plane) were presented. Colours from yellow to red indicate stress. The beige colour corresponds to zero or toward zero stress while the red colour indicates maximum stress. The total results of stress and strain were summarized in Tables 2 and 3. Figure 5 and 6 report the values of stress distribution in bones with high and low qualities.

**Table 2:** Values of maximum stress.

	4.2 × 13	4.2 × 13	4.2 × 13	4.2 × 13	4.2 × 13	4.2 × 13
	SFB Implant	SFB Implant	SFB Implant	SFB Implant	SFB Implant	SFB Implant
	Straight Abu	Straight Abu	15° Abu	15° Abu	25° Abu	25° Abu
	FY=200 N	FY=200 N	FY=200 N	FY=200 N	FY=200 N	FY=200 N
		FZ=140N		FZ=140N		FZ=140N
	(σ _MAX_ Von Mises)	(σ _MAX_ Von Mises)	(σ _MAX_ Von Mises)	(σ _MAX_ Von Mises)	(σ _MAX_ Von Mises)	(σ _MAX_ Von Mises)

D1 and D4	83	205	267.8	1025.5	246.3	671.8

**Table 3:** Values of maximum strain.

	4.2 × 13	4.2 × 13	4.2 × 13	4.2 × 13	4.2 × 13	4.2 × 13
	SFB Implant	SFB Implant	SFB Implant	SFB Implant	SFB Implant	SFB Implant
	Straight Abu	Straight Abu	15° Abu	15° Abu	25° Abu	25° Abu
	FY=200 N	FY=200 N	FY=200 N	FY=200 N	FY=200 N	FY=200 N
		FZ=140N		FZ=140N		FZ=140N
	(ε_MAX total micro-strain_)	(ε_MAX total micro-strain_)	(ε_MAX total micro-strain_)	(ε_MAX total micro-strain_)	(ε_MAX total micro-strain_)	(ε_MAX total micro-strain_)

D1	0.00107	0.00988	0.0053	0.0154	0.00558	0.01372
D4	0.00274	0.00911	0.0048	0.0148	0.00517	0.01269

## Discussion

Primary implant stability and bone density are variables which are considered essential to achieve predictable osseointegration and long-term clinical survival of implants.^15^ Zarb and Schmitt^16^ stated that bone structure is the most important factor in selecting the most favourable treatment outcome in implant dentistry. Bone represents the external architecture of the edentulous area considered for implants. In addition, it has an internal structure described in term of quality or density, which reflects the strength of the bone.^17^ For osseointegration of endosteal implants to occur, not only adequate bone quantity is required, but adequate density is also needed.^18^ The initial bone density not only provides mechanical immobilization of the implant during healing, but after healing also permits distribution and transmission of stresses from the prosthesis to the implant bone interface.^19^ The mechanical distribution of stress occurs primarily where bone is in contact with the implant.^17^ Williams et al^20^ demonstrated that when maximum stress concentration occurs in cortical bone, it is located in the area of contact with the implant, and when the maximum stress concentration occurs in trabecular bone, it occurs around the apex of the implant. In cortical bone, stress dissipation is restricted to the immediate area surrounding the implant; in trabecular bone, a fairly broader distant stress distribution occurs.^21^ Misch^17^ established that the percentage of bone contact is significantly greater in cortical bone than in trabecular bone. Cortical bone, having a higher modulus of elasticity than trabecular bone, is stronger and more resistant to deformation. For this reason, cortical bone will bear more load than trabecular bone in clinical situations.^22^ The classification scheme for bone quality proposed by Lekholm and Zarb^23^ has since been accepted by clinicians and investigators as standard in evaluating patients for implant placement. In this system, the sites are categorized into 1 of 4 groups on the basis of jawbone quality. In Type 1 (D1) bone quality, the entire jaw is comprised of homogenous compact bone. In Type 2 (D2) bone quality, a thick layer (2 mm) of compact bone surrounds a core of dense trabecular bone. In Type 3 (D3) bone quality, a thin layer (1 mm) of cortical bone surrounds a core of dense trabecular bone of favorable strength. In Type 4 (D4) bone quality, a thin layer (1 mm) of cortical bone surrounds a core of low-density trabecular bone.^17^,^23^–^26^ With the use of 3-dimensional FEA, Sevimay et al^15^ investigated the effect of these 4 different bone qualities on stress distribution in an implant-supported mandibular crown. They showed the presence of lower stresses for D1 and D2 bone qualities and increased stresses for D3 and D4 bone qualities because the trabecular bone was weaker and less resistant to deformation. Bone is a porous material with complex microstructure. It is an anisotropic material, which means it has different physical properties when measured in different directions.^1^ Canay et al^27^ compared vertically orientated implants with angled implants and found that the inclination of implants greatly influences stress concentrations around the implants. In the present study, a 3D FEA was performed to analyze the stress distribution within two different bone types (i.e., high and poor qualities) due to forces applied to three implant systems (i.e., one spiral implant plus one straight abutment, or one 15 degree angulated abutment, or one 25 degree angulated abut-ment). The minimum bone stress was produced with straight abutment and vertical force whereas the maximum bone stress was obtained with 15 degree angulated abutment and coupled forces. In addition, we found the lower the bone quality (i.e., D4), the higher the distribution of the stress within the bone.

## Conclusion

In conclusion, spiral family implants can be used successfully in low bone quality but a straight force is recommended.
